# Invisalign Treatment of a Three-Year-Old Child with Bilateral Posterior Scissor Bite and Multisite Upper Airway Obstruction: A Case Report

**DOI:** 10.3390/jcm12010333

**Published:** 2023-01-01

**Authors:** Yilin Xin, Hongling Zhou, Yifan Zhao, Lixing Zhao

**Affiliations:** State Key Laboratory of Oral Diseases, National Clinical Research Center for Oral Diseases, Department of Orthodontics, West China Hospital of Stomatology, Sichuan University, Chengdu 610041, China

**Keywords:** pediatrics, orthodontic, malocclusion, primary dentition, scissor bite, crossbite, Invisalign aligners, airway obstruction, multidisciplinary

## Abstract

Background: Scissor bites have been reported in relatively few epidemiological studies because of their extremely low prevalence rate (below 1%). The etiology of scissor bites remains obscure, but its impact on growth and function should not be ignored. Methods: In this case report, a novel treatment that utilizes Invisalign aligners was performed on a 3-year-old child who presented with a bilateral posterior scissor bite and anterior crossbite, accompanied by multisite obstruction in the upper airway. The aligners functioned as occlusion pads to unlock the scissor bite relationship and combined with cross-traction to narrow the maxillary arch and enlarge the mandibular arch simultaneously. Results: The duration of orthodontic therapy was 28 weeks. A multidisciplinary consultation (orthodontics department, ENT department, and spinal surgery) was conducted and a stable result was achieved. A healthy occlusal relationship, improved dental esthetics and a better lateral profile were eventually obtained. Conclusions: Positive treatment outcomes rely on patients’ good compliance in this case. In addition, we hope that clinicians will consider our situation in terms of alternative treatments and interprofessional experience.

## 1. Introduction

A scissor bite is defined as a malocclusion where the maxillary molars occlude the buccal surfaces of the corresponding mandibular molars and/or mandibular molars occlude the lingual surfaces of the counterparts. It reflects the discrepancy of the dental arches or the skeletal disharmonies, and it was estimated that 1.5% of the general population and even fewer children are affected by this anatomical variation [1]. Moreover, the reported incidence might be underestimated, as the afflicted patients often disregard it, since no obvious facial alterations appear in the early periods. The precise etiology of the scissor bite remains obscure. However, its adverse impact should never be neglected. On the one hand, it can weaken the patient’s masticatory function and impede the growth and development of children. On the other hand, it can disrupt the dynamic musculature equilibrium during the mandibular excursion, resulting in abnormalities in the temporomandibular joint (TMJ) and other problems.

Based on Moss’s functional matrix theory, the mandible, its associated muscle groups and TMJ are viewed as an interrelated organic whole, whose growth and development respond to specific functional requirements [2]. In addition, respiration, an essential function of the craniomaxillofacial system, also determines the morphology of the hard and soft tissues that surround the upper airway [2,3]. Once the upper airway is obstructed, the body will compensate for the deficiency in normal nasal ventilation. For instance, to maintain regular ventilation, mouth breathing, a common oral habit that alters the posture of the head and tongue or even the hyoid bone, may be necessary. Hence, variations in the respiratory pattern may result in different types of malocclusion and bring changes to maxillofacial development, head position and cervical curvature [4,5,6].

There are several traditional ways to treat scissor bites, such as fixed or removable appliances or surgical approaches. In this study, we utilize Invisalign aligners, which are different from conventional appliances in some aspects, to treat a scissor bite in the primary dentition. This is because it has been shown that removable clear aligners facilitate tooth cleaning, and reduce the incidence of white spot lesions and gingivitis, as well as periodontitis [7,8]. In addition, compared to patients who have fixed orthodontic appliances, plaque accumulation and periodontal risk are lower in individuals who undergo clear aligner therapy. In addition, owing to the existence of increased sites for bacterial adhesion and biofilm development (plaque accumulation), fixed appliances contribute to inflammation and difficulties in achieving adequate oral hygiene [9,10].

Given the aforementioned theory, early detection, precise diagnosis and proper correction of scissor bites, as well as airway obstructions, are of tremendous clinical significance in orthodontic therapy, especially for pediatric patients. This report aimed to describe a relatively rare case of a 3-year-old child with an anterior crossbite and bilateral posterior scissor bite, which was accompanied by a multisite obstruction in the upper airway.

## 2. Materials and Methods

### 2.1. Diagnosis and Etiology

A 3.5-year-old male patient sought orthodontic treatment at our hospital with his parents, whose chief complaints were difficulty chewing food with his posterior teeth and protruding lower anterior teeth. No other systemic disorders were reported during the appointment.

The boy had a complex medical history. First, he had bad sleeping posture, prone to sleeping on his stomach since infancy. In addition, when feeding, he adopted an abnormal bottle-feeding position, lying down, which may result in habitual mandibular protraction. At 1 month old, he was diagnosed with atopic dermatitis (AD) because of his widespread itching, eczema, and allergic rhinitis (AR). Around 8 to 9 months old, he started mouth breathing and snoring intermittently. In addition, his upper gingival area was accidentally injured at the age of 1.5 years old, leading to a forward mandible position, unconsciously moving forward to protect the maxillary soft tissue. A habit of tongue flicking during sleeping was also reported. No pronounced familial history of skeletal Class III or II malocclusion was reported.

**Figure 1:**
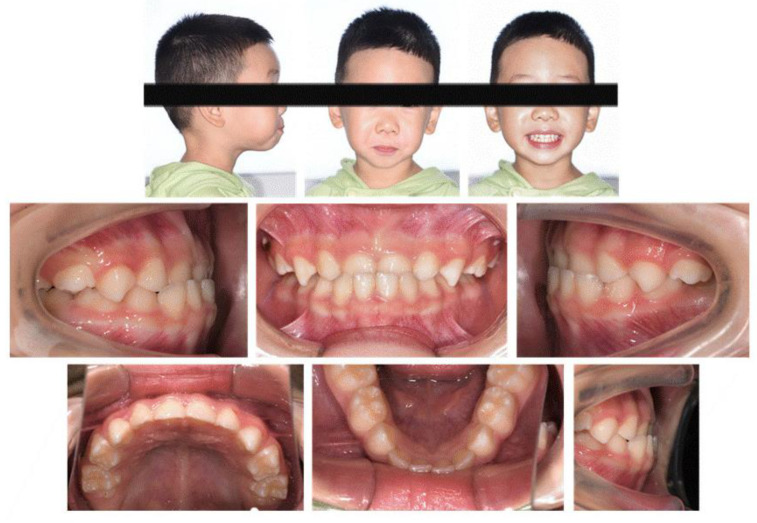
Initial photographs.

**Figure 2:**
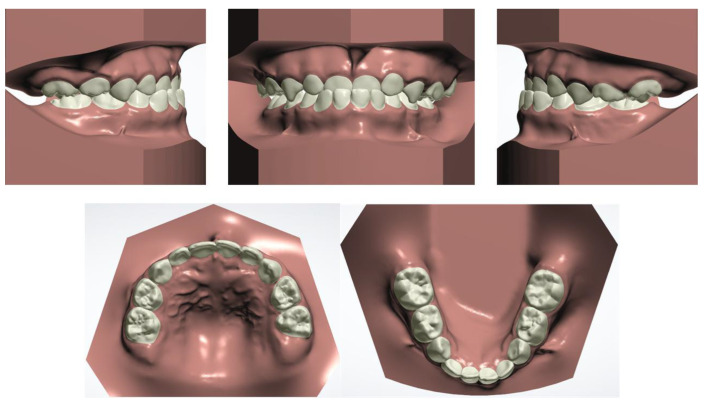
Initial digital study models.

**Figure 3:**
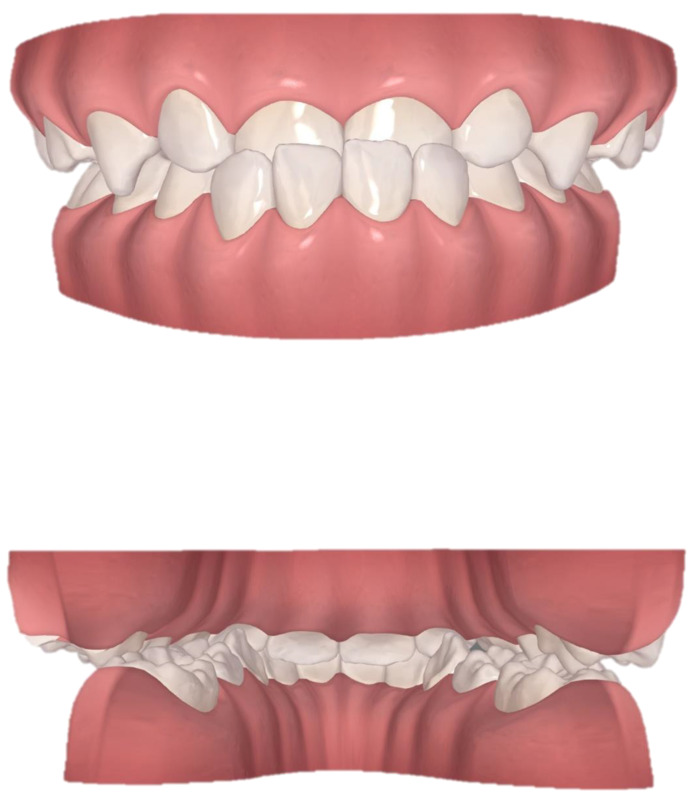
View of scissor bite details of the initial digital models.

The clinical examination revealed a convex profile with protrusion of the upper and lower lips and hypertonia of the lip muscles (Figure 1). An intraoral examination identified a primary dentition phase. Centric occlusion examination indicated a Class III terminal molar relationship with a bilateral posterior scissor bite. The left posterior teeth presented a complete scissor bite with no contact on the occlusal surface, which was worse than their right counterparts (Figure 2 and Figure 3). An anterior crossbite was observed with a reverse overjet and overbite. Furthermore, the jaw could be retracted to display an edge-to-edge bite when guided backward. The mandibular incisors were upright, with the upper incisors slightly lingually inclined. The upper and lower dental midlines were aligned with the facial midline. No functional mandibular shift was observed when the mouth was open, and no clicking or pain was detected in the TMJ area. Moreover, habitual mandibular protraction at rest and during speech was observed.

**Figure 4:**
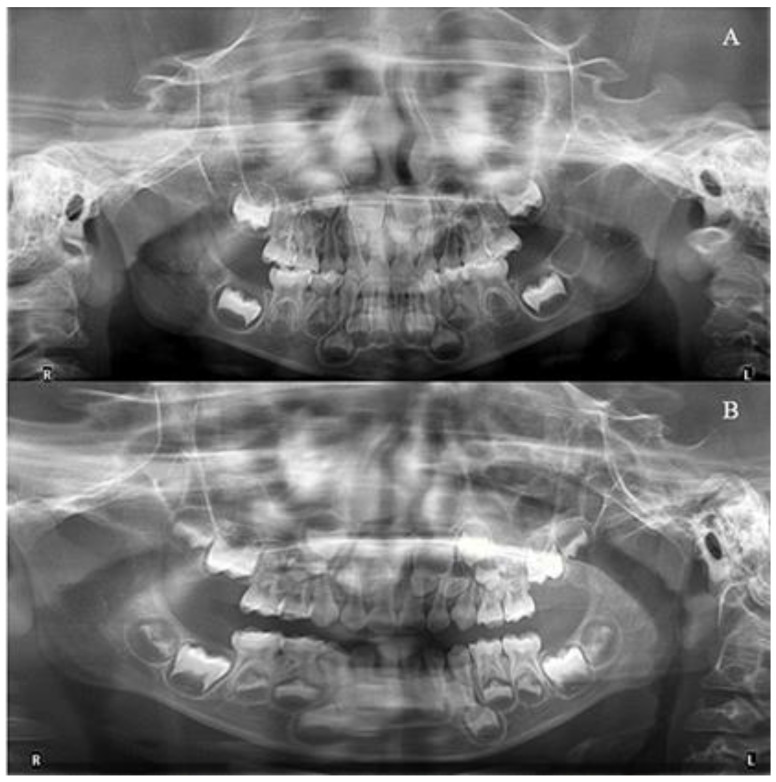
The initial and final radiographs. (**A**): Pre-treatment; (**B**): Post-treatment.

**Figure 5:**
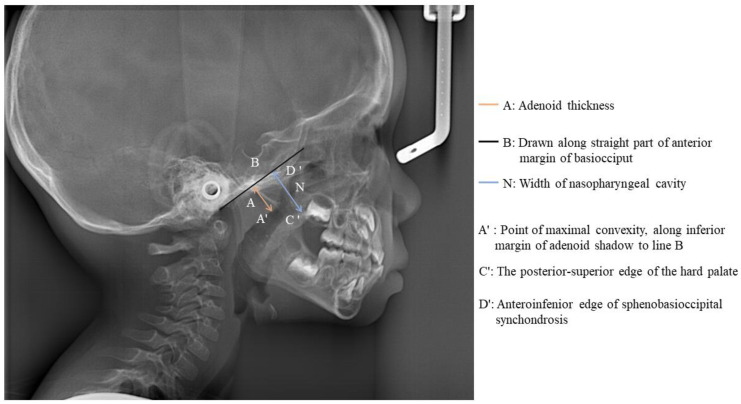
Measurement of adenoid thickness.

**Figure 6:**
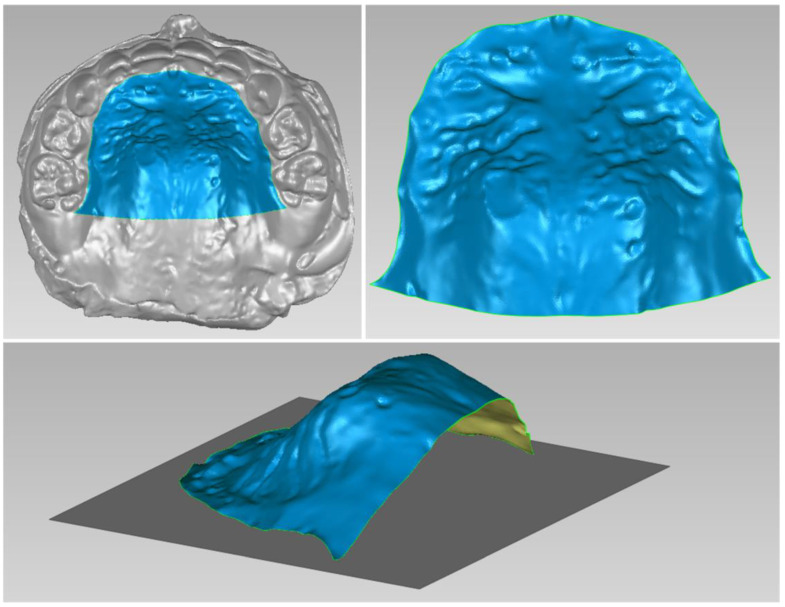
Digital cast measurement of the initial cast.

**Figure 7:**
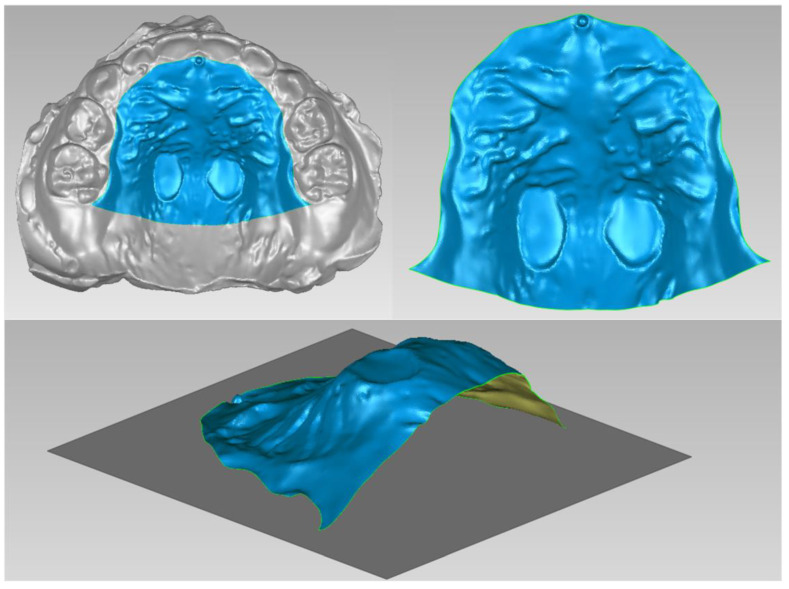
Digital cast measurement of the final cast.

The patient and his parents allowed the use of his medical records, photographs and radiographs in this research. Prior to the initiation of therapy, panoramic and lateral cephalometric films were obtained. The panoramic radiograph showed swelling in the bilateral nasal mucosa (Figure 4). A mild skeletal Class II sagittal relationship (ANB = 5.4°) and large mandibular plane angle (SN-MP = 39.8°) were indicated in the pre-treatment cephalometric film (Table 1). Meanwhile, his hyoid (MP-H) rested lower than normal (16.8 mm, mean normal = 14.2). In addition, he had a hyperdivergent growth pattern (facial height index, S-Go/N-Me = 57.9%). The airway width assessment showed that he had a limited nasopharyngeal airway gap and expanded oropharyngeal segments (data shown in Table 1). His adenoids were diagnosed with moderate hypertrophy (Figure 5) with an A/N of 64.5% [11,12], which was derived from an adenoidal–nasopharyngeal ratio (AN ratio) via linear measurements using lateral radiographs of the nasopharynx according to Fujioka et al. and Zou. et al. [11,12]. Considering that adenoids in children are physiologically enlarged between 2 and 12 years of age, Zou proposed that an A/N ratio of 0.60 should be considered as normal; 0.61–0.70 is considered as moderate hypertrophy; ≥0.71 is considered as pathological hypertrophy [12]. The digital models’ STL files were uploaded to the Geomagic Studio 12.0 software (Research Triangle Park, NC, USA) for measurement (Figure 6 and Figure 7). The statistics concerning the widths of the dental arch and basal skeletal, as well as the height, surface area and volume of the palatal, are displayed in Table 2.

**Figure 8:**
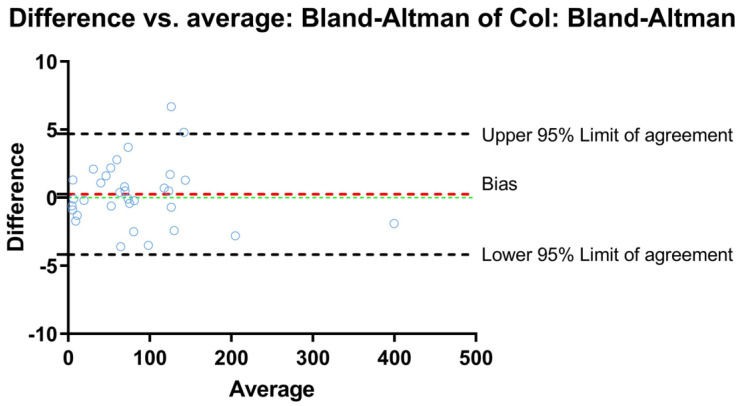
Bland and Altman plot for data measured over 2-week intervals concerning cephalometric analysis.

One operator assessed the reproducibility of the results by calculating intraclass correlation coefficients (ICC) for two repeated measurements within 2-week intervals. The ICC value was 0.985, with a 95% confidence limit (CI) of 0.991, which was calculated from the values of 0.847 and 0.998 (Figure 8).

### 2.2. Treatment Objectives

The following treatment objectives were established: (1) correct the scissor bite, (2) adjust the anterior crossbite to achieve a normal overjet and overbite and also correct the habitual mandibular protraction, (3) eliminate the CR–CO discrepancy and obtain a stable occlusal relationship, (4) improve dental and facial esthetics, (5) carry out multidisciplinary treatment of adenoid hypertrophy (AH) with the ENT department to eliminate the causes of malocclusion as much as possible, and enable improved skeletal face growth and development.

### 2.3. Treatment Alternatives

Correcting the posterior scissor bite was crucial in achieving the aforementioned treatment objectives. Two options were considered to achieve the main objective. The first treatment option was to use a constriction quad-helix appliance bonded to the maxillary arch to limit the arch width, as bi-helix mandibular appliances promote the development of the arch width. In addition, the hyperbolic reeds attached to the quad-helix on the maxilla that were used to adjust the anterior crossbite were simultaneously indispensable. However, compared with permanent teeth, the small amount of primary molars was more likely to cause the detachment of the appliance, and thus result in an inferior and unstable outcome. Meanwhile, it may also bring about complications such as caries or mucosal damage. Given this, the parents did not take this method into account.

The second plan was to alleviate the occlusion discrepancy of the dental arch with Invisalign aligners. The aligners function as occlusion pads to unlock the scissor bite relationship and combine with cross-traction to narrow the maxillary arch and enlarge the mandibular arch simultaneously. In this method, uncontrolled mild extrusion of the anchorage teeth may occur. However, the sense of comfort when wearing Invisalign could increase patient compliance and avoid damage to the mucosa. Therefore, considering the above factors, we agreed with his parents to choose this option. Then, we used stepwise intrusion of the lower anterior teeth and modest proclination of the upper anterior teeth to resolve the anterior crossbite. We also attempted to prevent mouth breathing with an anti-snoring patch after curing rhinitis.

### 2.4. Treatment Progress

**Figure 9:**
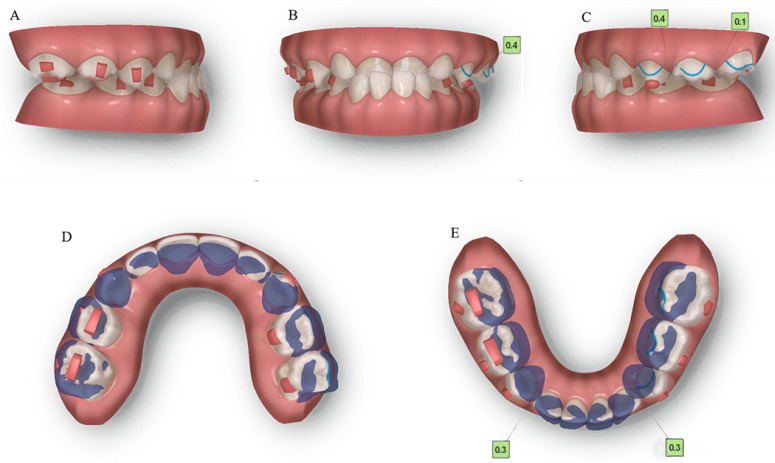
Pre-treatment ClinCheck^®^ models and ClinCheck^®^ treatment plan. (**A**): Lateral view of the right side of the pre-treatment model; (**B**): frontal view of pre-treatment model; (**C**): lateral view of the left side of the pre-treatment model; (**D**): occlusal view of superimposition of pre-treatment (blue) and post-treatment (white) maxillary arch models; (**E**): occlusal view of superimposition of pre-treatment (blue) and post-treatment (white) mandibular arch models.

We used ClinCheck^®^ (Align Technology, San Jose, CA, USA) to plan tooth movement virtually in three dimensions (depicted in Figure 9). The ellipsoid and optimized attachments on teeth 63 and 85 promoted retention. The rectangular attachments on the occlusal surfaces of teeth 54, 55, 84 and 85 increased the vertical height to unlock the scissor bite. On the left side, the extent of the scissor bite was more severe than on the right side; therefore, we placed rectangular attachments on the lingual surfaces of the upper left deciduous molars and buccal surfaces of the lower molars. Meanwhile, a button cutout was designed for the upper left buccal and lower lingual deciduous molars, utilizing the interaction traction with elastics. Overall, the upper posterior segments moved lingually, while the lower counterparts moved buccally to resolve the dental arch discrepancy, and thus alleviate the scissor bite. Furthermore, the upper anterior teeth were proclined, while the lower teeth progressively moved to alleviate the anterior crossbite, placing the bilateral condyles into centric relation.

**Figure 10:**
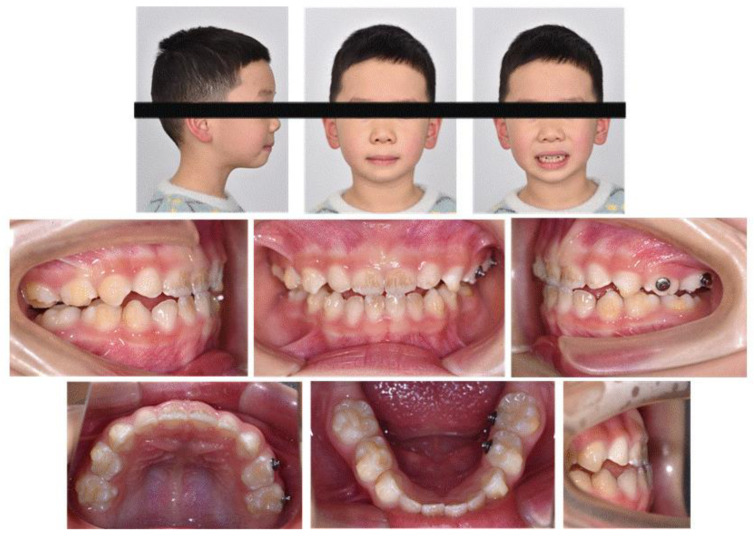
Intraoral photograph after the 8th aligner.

**Figure 11:**
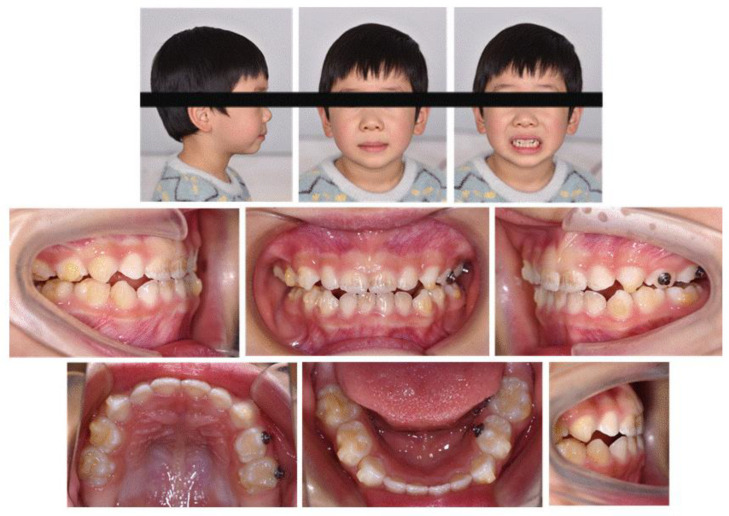
Intraoral photograph after the 20th aligner.

We instructed the patient to wear each aligner continuously for 22 h daily and scheduled a monthly checkup. A total of 28 aligners were designed, and a program that required changes every seven days was implemented. Neither cases of detachment nor complaints of pain, discomfort, or impairment of function were reported. When the patient wore the 8th aligner, the anterior crossbite was corrected with convex profile improvement (Figure 10). With the 20th aligner, the posterior scissor bite was also corrected by maxillary narrowing and mandibular widening (Figure 11). Until the 25th aligner, nearly all of the chief complaints were settled, except for the insufficient tightness of occlusion on the right posterior teeth.

**Figure 12:**
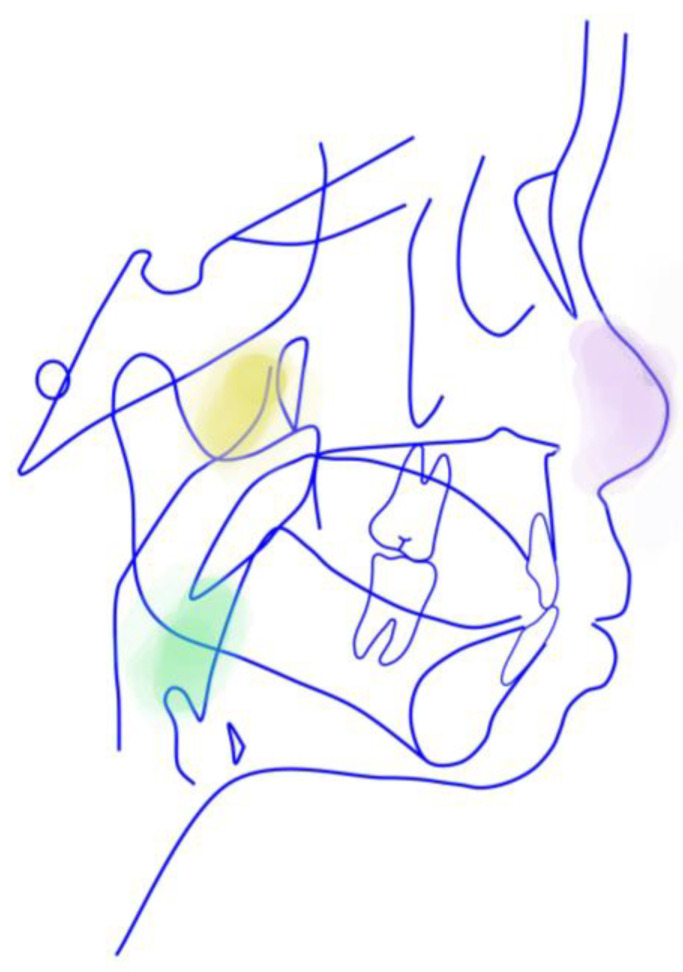
Schematic of upper airway multisite obstruction clinical manifestations.

**Figure 13:**
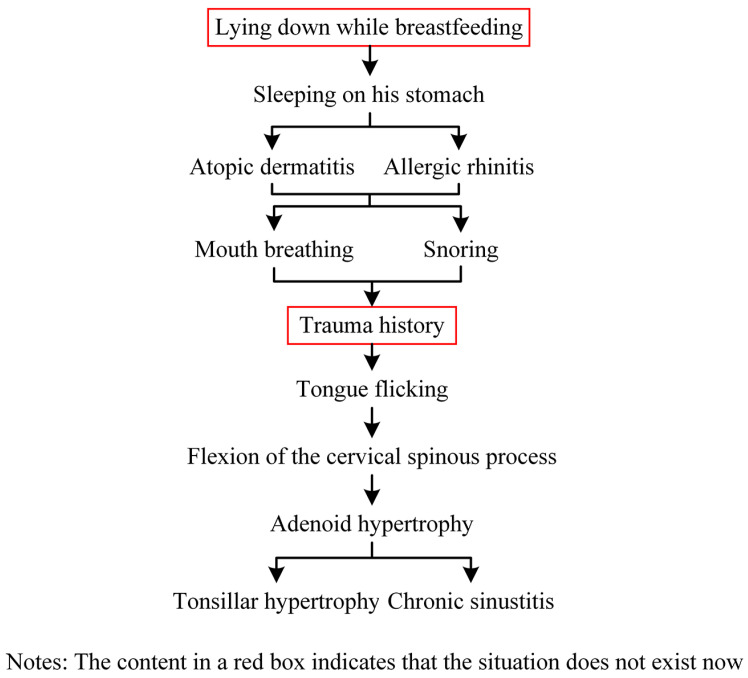
The whole timeline of the disease history.

**Figure 14:**
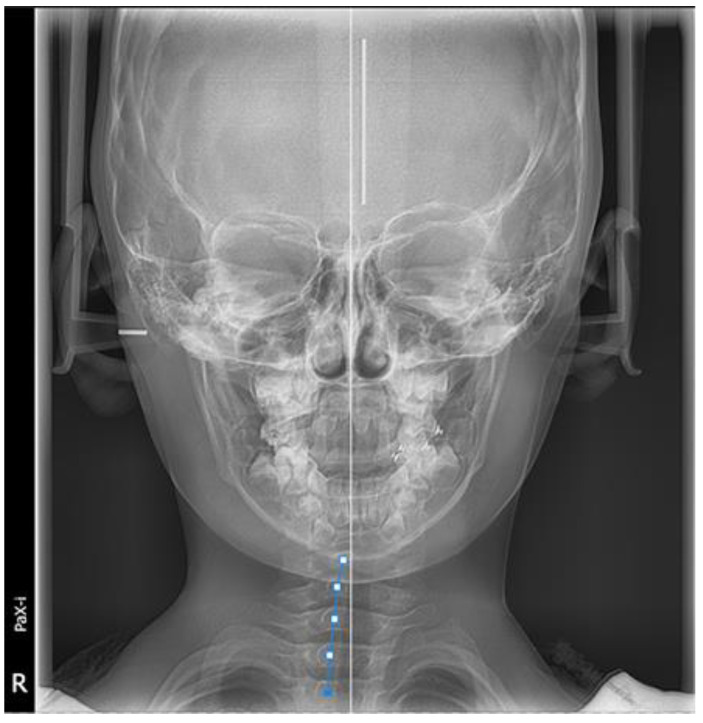
Intraoperative posteroanterior cephalograms.

**Figure 15:**
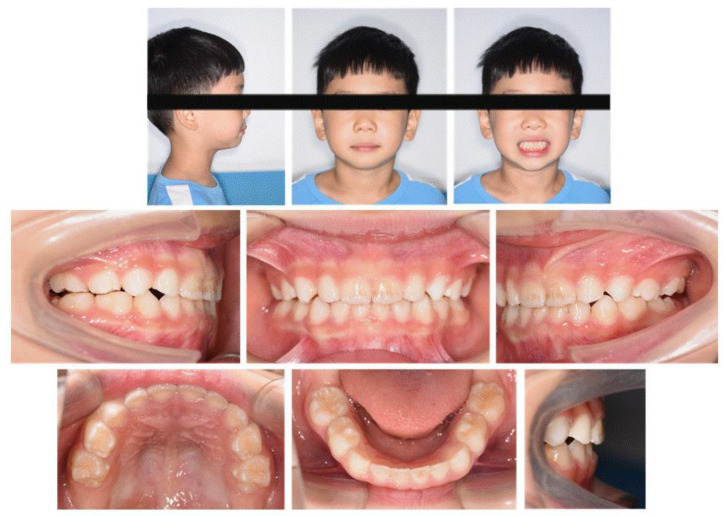
Final photographs.

**Figure 16:**
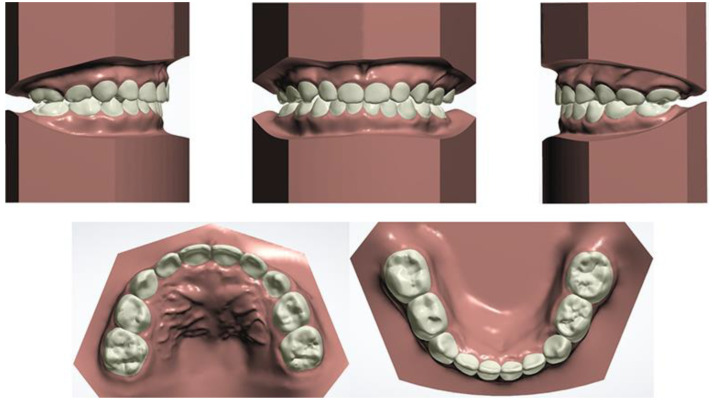
Final digital study models.

Furthermore, during orthodontic treatment, his parents visited the ENT department for the hoarseness of the boy’s vocal cords. Rhinoscopic examination revealed enlarged adenoids that blocked 1/4 of the posterior nostril and bilateral vocal fold nodules. The boy was also diagnosed with second-degree tonsillar hypertrophy, variable rhinitis and chronic sinusitis (schematic in Figure 12; the whole timeline of disease history in Figure 13). The otolaryngologist prescribed nasal drops for rhinitis and suggested a follow-up for other diseases, including tonsillar and adenoids. After treatment, the bilateral nasal mucosa swelling was alleviated, as indicated in Figure 3. Moreover, the intraoperative posteroanterior cephalograms revealed a mild flexion of the cervical spinous process, which required observation and follow-up according to a spine surgeon (Figure 14). The therapy duration was seven months, after which all the chief complaints were resolved (Figure 15 and Figure 16).

## 3. Results

**Figure 17:**
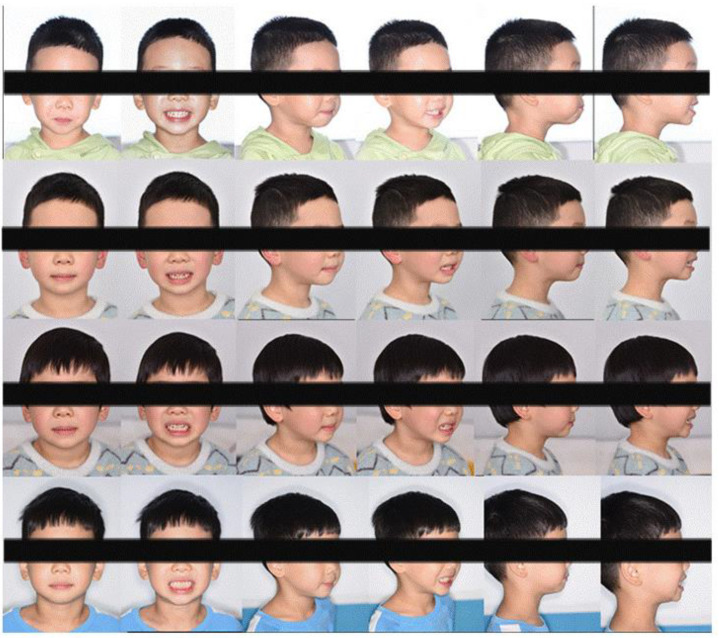
Changes in lateral profile.

**Figure 18:**
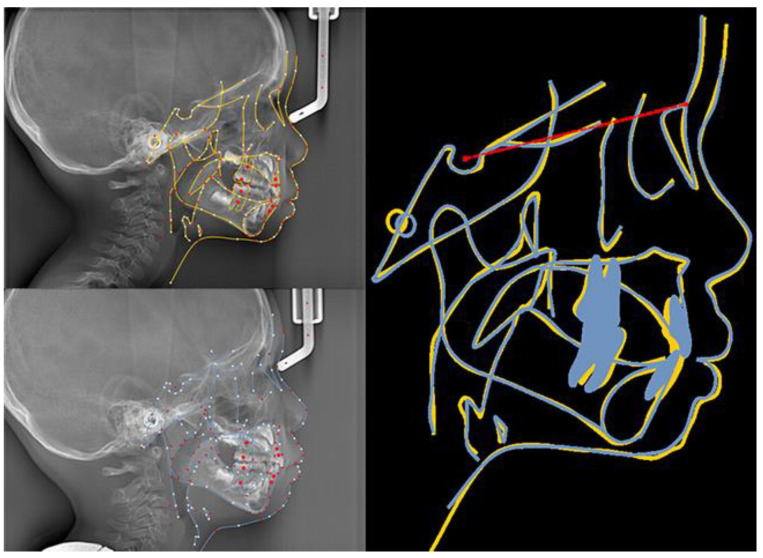
The initial and final lateral cephalograms and overlapping graphs of cephalometric tracing.

A total of 28 aligners were used during the whole treatment period. A healthy occlusal relationship, improved dental esthetics and a better lateral profile were eventually obtained. Following the narrowing of the upper dental arch and enlargement of the lower dental arch, the maxillary and mandibular widths were matched. Mild mandibular molar distalization and maxillary molar mesialization were used to acquire an ideal canine and molar sagittal relationship by correcting the bilateral posterior scissor bite and anterior crossbite. By adjusting the angle of the incisors, we achieved the optimal overbite and overjet for the patient. Esthetic improvement and a relatively straight profile were also achieved due to his labially inclined upper incisors from the lateral view (Figure 17). Figure 18 depicts the initial and final lateral cephalograms. No recurrence of the anterior crossbite or bilateral posterior scissor bite was observed during the 3-month follow-up.

## 4. Discussion

The low incidence of scissor bites has led to fewer reports concerning its treatment. Traditional scissor bite treatments often involve surgical procedures and fixed appliances. However, some people do not consent to surgical therapy due to its invasiveness, expense and related dangers. In addition, fixed appliances such as maxillary constriction plates, intermaxillary elastics, quad-helix and transpalatal arch appliances can also be applied. However, with these appliances, it is more difficult to maintain meticulous oral hygiene, they are more likely to injure the soft tissues of the mouth, and have less aesthetic and concealing benefits [13,14]. Among them, a quad-helix is not a bad alternative, since the reported benefits include minimal discomfort, less requirement for patient cooperation, improved control over tooth movements and cost-effectiveness.

However, we utilized aligners in this case for two reasons. On the one hand, aligners have demonstrated the aforementioned therapeutic benefits and wear comfort [13,15]. On the other hand, the thickness of aligners combined with the attachments on the occlusal surface may serve as an occlusion pad to release the locking of the occlusion. In this way, we make use of the advantage of traditional removable appliances, while avoiding the downsides of fixed devices. However, the limits of Invisalign aligners should also be noted. Compliance with treatment must be carefully examined and only patients with excellent compliance may obtain positive outcomes. Nevertheless, studies have suggested that younger patients may have a higher percentage of treatment compliance, as the noncompliance rate for twin-block therapy was 16% at 9.7 years old and 33.63% at 12.4 [16,17]. Therefore, early treatment of severe malocclusions using detachable equipment may have a good likelihood of success.

**Figure 19:**
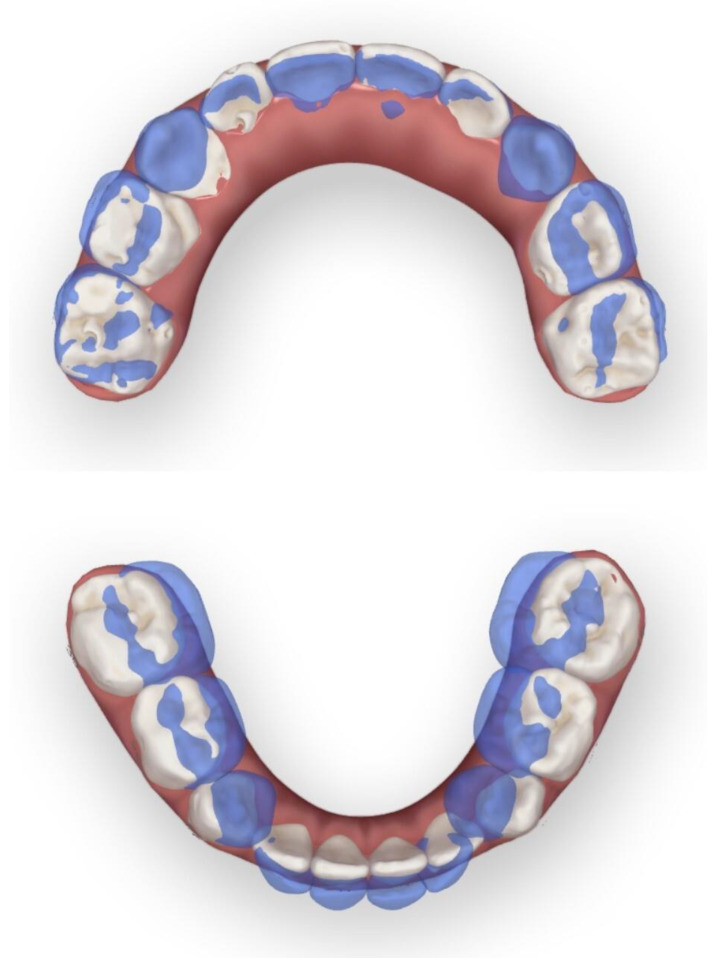
Initial and final model overlay contrast.

The data of treatment outcomes are displayed in Table 2, with regard to dental arch widths, basal skeletal widths, palatal volume and surface area. In contrast with the initial and final model in Figure 19, mandibular arch narrowing following maxillary and arch expansion was achieved by Invisalign aligners. Moreover, the intricate relationships between a 3-year-old boy’s malocclusion, airway and the rest of his body were of significant interest. Pediatricians, otorhinolaryngologists, orthodontists and other professionals have long been interested in the association between respiratory patterns and facial skeletal development [18,19].

**Figure 20:**
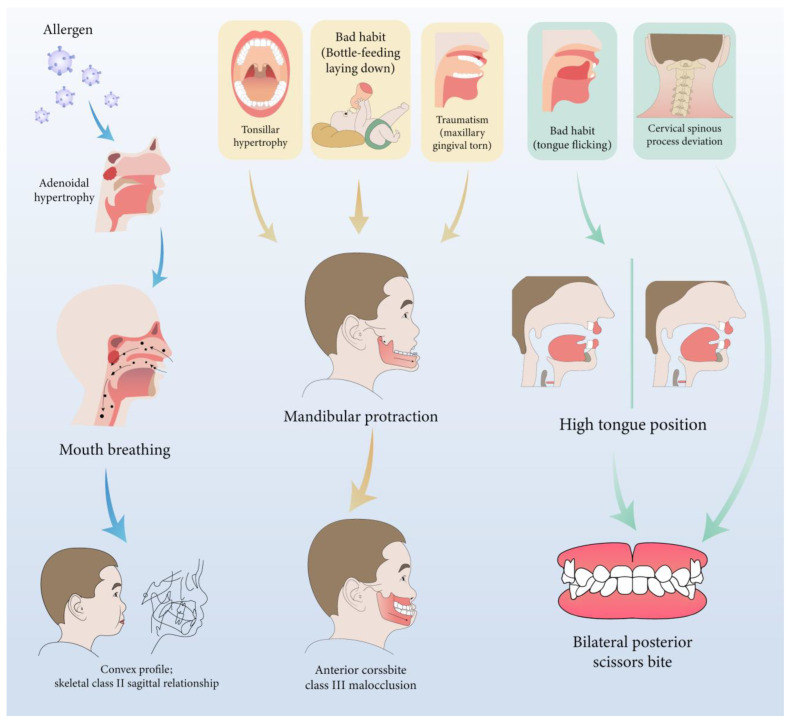
The inference diagram of the relationship between etiology, mechanism and clinical manifestations.

Since the etiology of scissors bites is still obscure, we intend to discuss the intersection of multidisciplinary knowledge here. We noticed complex symptoms in this patient following no further intervention treatment, except for malocclusion and nasal mucosa treatment, because his symptoms were not too severe to be treated in the tonsillar, adenoids and cervical spinous process. In addition, during the multidisciplinary consultation, we found a lack of awareness of the etiology and influence of the scissor bite and connection with other parts of the body among ENT surgeons and spine surgeons. Overall, the boy’s early malocclusion and craniofacial morphology changes could be attributed to the following two environmental factors: pathological breathing patterns (multiple obstructions in the upper airway) and poor habits. Furthermore, we also tried to analyze the relationship shown in Figure 20. The discussion focused on the following three dimensions: sagittal, vertical and coronal.

From the sagittal perspective, multisite airway obstructions contributed to the condition of the mild skeletal Class II sagittal relationship in this patient. Any obstacle in the nasopharyngeal cavity, such as anatomical predisposition or pathologies including palatine and pharyngeal tonsils hypertrophy, AR and nasal turbinate hypertrophy, could result in respiratory obstruction, and thus force the patient to breathe through their mouth [20,21,22]. Allergic diseases (AR, bronchial asthma or AD) contribute to a higher prevalence (40.4%; 22.3% for the control group in another study) of AH in children [23]. Metaplasia may be a risk factor for AH, which could be the initial etiology of this patient [24]. In addition, allergic factors also contribute to the sagittal underdevelopment of the maxillofacial region, embodying the value of SNA and SNB decreases [25]. Moreover, tonsils, which occupy a significant portion of the oropharyngeal space, may dictate the anterior tongue position in this study, with the tongue exerting pressure on the anterior region of the mandible [6,26]. In this case, it tends to result in an anterior crossbite and Class III molar relationship.

Vertically, mouth breathing also leads to a lower position of the mandible, which is usually associated with lower orofacial muscle tonicity [27]. Solow and Kreiborg suggested that obstruction causes contraction of the soft tissues of the face and pharynx on the dorsal side. It restricts the jaws from developing forward, resulting in posterior downward rotation of the mandible.

From the coronal perspective, tongue size, posture and function are of crucial importance in the etiology of malocclusions and dentofacial deformities. The transverse dimension of the maxillary arch is negatively correlated with the tonsillar grade [5]. This reduction may be attributed to the tongue’s lowered position in patients with higher grades, who are more susceptible to ventilatory disturbance [5]. Tongue posture has also been linked to increased mandibular arch width [28,29]. Interestingly, a wider maxillary arch and higher tongue posture exist in this case. The discrepancy in dental arches may result from the prolonged duration of high lingual posture, the more robust lingual muscle strength and the disrupted orofacial muscle balance. Furthermore, combined with obstruction in the upper airway, mouth breathing, as a compensation method, has to increase the air intake volume to meet the demand. Therefore, due to the combined influence of the buccal muscle and airflow, the lower dental arch ultimately becomes smaller, resulting in a scissor bite.

Moreover, the intraoperative posteroanterior cephalograms of the patient revealed a mild deviation in the spinal spinous process (Figure 14), which may relate to the pathogenesis of the scissor bite. A study also suggested that thoracic scoliosis was correlated with scissor bites and asymmetry in the area of the brachial band, leading to compensatory scoliosis of the cervical spine and hyper-kyphosis in the thoracic spine [30]. As the etiology of the scissor bite remained unclear, we speculated that it might serve as a cause for the malocclusion in this case.

Here, we analyzed the relationship between upper airway factors, muscle chains, spine, and maxillofacial morphology, whose cause and effect should be interpreted with care. This article represents an initial exploration of this area and further study could be conducted in this multidisciplinary field in the future.

Moreover, this patient was more compliant than the average child, resulting in a smooth treatment course. In addition, the latest technology, Biorepair Parodontgel, could be used with aligners at home in the future to enhance dental hygiene and improve Invisalign aligner therapy results. It may have an anti-inflammatory and regenerative impact on periodontal tissues and cause remineralization of the root surface of teeth [31].

The limitation of this research was the lack of myofunctional training that could be simultaneously combined with Invisalign aligners. Frey and Maspero et al. agreed that according to the treatment mechanism of respiratory disorders and abnormal swallowing, emphasis should be placed on the inclusion of functional orthodontic aids for oral muscles [32,33]. This includes the treatment of facial muscle imbalances, training of the tongue posture, and establishing an equilibrium between the tongue, lip, and cheek muscles. Myofunctional exercise could also be incorporated to maintain good development and a suitable growth environment [34,35]. The aforementioned technology and training could be utilized in future medical treatments and scientific studies.

## 5. Conclusions

This case illustrates the effectiveness of Invisalign aligners in correcting anterior crossbites and bilateral posterior scissor bites in the primary dentition, achieving a favorable and stable result. Aligner treatment offers a method for correcting complex cases of deciduous dentition. However, good compliance was essential for this device. Myofunctional training should also be combined with Invisalign aligner treatment in further treatment and research. Additionally, the interdisciplinary cooperation between orthodontists, ENT specialists, and spine surgeons was strengthened. Early diagnosis and treatment provide a favorable environment for growth and development.

## Figures and Tables

**Table 1:** Cephalometric analysis.

Variables	Norm	Pre-Treatment	Post-Treatment	Change
**Angular (°)**				
SNA	82.0 ± 3.0	75.0	76.2	1.2
SNB	78.0 ± 3.0	69.7	70.8	1.1
ANB	3.0 ± 2.0	5.4	5.3	−0.1
Na-S-Ar	123.0 ± 5.0	126.8	125.5	−1.3
Ar-Go′-Me	123.0 ± 7.0	125.9	126.9	1.0
GoGn-SN	31.2 ± 3.6	38.6	40.8	2.2
FH-NPo	85.0 ± 4.0	79.5	80.9	1.4
FH-MP	26.0 ± 4.0	29.6	33.0	3.4
SN-MP	30.0 ± 6.0	39.8	44.8	5.0
SN-OP	19.0 ± 4.0	34.1	25.9	−8.2
MP-OP	11.0 ± 5.0	5.8	19.0	13.2
U1-SN	106.0 ± 6.0	80.2	91.3	11.1
L1-FH	55.0 ± 2.0	52.2	62.7	10.5
L1-MP	97.0 ± 6.0	98.2	84.4	−13.8
U1-L1	124.0 ± 8.0	141.9	139.6	−2.3
**Linear (mm)**				
S-N	71.0 ± 3.0	81.1	63.4	−17.7
N-Me	112.0 ± 7.0	123.3	100.3	−23.0
N-Go′	95.0 ± 4.0	124.9	99.7	−25.2
S-Go′	80.0 ± 6.0	73.4	59.6	−13.8
*Y*-axis length	86.0 ± 6.0	117.7	94.6	−23.1
LL-EP	1.0 ± 2.0	6.5	3.0	−3.5
UL-EP	−1.0 ± 1.0	4.5	4.0	−0.5
Wits	0.0 ± 2.0	−4.7	1.0	5.7
PNS-R	25.5 ± 3.0	16.8	19.0	2.2
PNS-UPW	31.4 ± 4.2	22.8	22.3	−0.5
U-MPW	11.5 ± 2.9	15.9	14.4	−1.5
PAS (TB-TPPW)	13.5 ± 3.4	25.1	15.1	−10.0
V-LPW	17.5 ± 3.3	12.2	17.5	−1.2
SPT (SPP-SPA)	9.7 ± 1.6	17.4	11.4	−6.0
H-MP	9.7 ± 4.5	16.8	11.0	−5.8
H-CVP	39.6 ± 3.5	30.6	24.1	−6.5
**Ratio (%)**				
FHI (S-Go/N-Me)	63.0 ± 2.0	57.9	56.0	−1.9

**Table 2:** Statistics concerning initial and final digital casts.

Variables	Pre-Treatment	Post-Treatment	Change
**Dental arch widths (mm)**			
Maxillary			
Intercanine	31.9	33.0	1.1
Intermolar	44.8	43.3	−1.5
Mandibular			
Intercanine	21.4	25.4	4.0
Intermolar	39.2	45.6	6.4
**Basal skeletal widths (mm)**			
Maxillary			
Intercanine	38.7	37.0	−1.7
Intermolar	55.7	53.5	−2.2
Mandibular			
Intercanine	27.9	30.4	2.5
Intermolar	46.5	48.8	2.3
**Depth of the palatal vault (mm)**			
Maxillary	10.7	11.5	0.9
**Palatal volume (mm^3^)**			
Maxillary	4020.6	3412.3	−608.3
**Palatal surface area (mm^2^)**			
Maxillary	1034.2	1015.6	−18.6

## Data Availability

The data that support the findings of this study are available upon request from the corresponding author. The data are not publicly available, due to privacy or ethical restrictions.
